# Impact of 2 different posterior screw fixation techniques on primary stability in a cervical translational injury model: A biomechanical evaluation

**DOI:** 10.1097/MD.0000000000028866

**Published:** 2022-02-18

**Authors:** Ludwig Oberkircher, Julia Riemenschneider, Martin Bäumlein, Tom Knauf, Christopher Bliemel, Steffen Ruchholtz, Antonio Krüger

**Affiliations:** a Center for Orthopaedics and Trauma Surgery, Philipps University of Marburg, University Hospital Giessen and Marburg GmbH, Location Marburg, Germany; b Department of Trauma Surgery, Orthopaedics, Spine Surgery and Pediatric Trauma Surgery, Asklepios Hospital Lich, Lich, Germany.

**Keywords:** biomechanical, cervical, fracture, lateral mass screw, luxation, pedicle screw, spine

## Abstract

**Background:**

In case of injuries to the subaxial cervical spine, especially in osteoporotic bone, the question of the most stable operative technique arises. There are several techniques of screw fixation available regarding dorsal stabilization. This study investigates 2 techniques (lateral mass screws (LMS) vs cervical pedicle screws (CPS)) in the subaxial cervical spine regarding primary stability in a biomechanical testing using a translational injury model.

**Methods:**

A total of 10 human formalin fixed and 10 human fresh-frozen specimens (C 4 - T 1) were investigated. Specimens were randomized in 2 groups. Fracture generation of a luxation injury between C 5 and C 6 was created by a transection of all ligamentous structures as well as the intervertebral disc and a resection of the facet joints.

Dorsal stabilization of C 4/C 5 to C 6/C 7 was performed in group A by lateral mass screws, in group B by pedicle screws. In the biomechanical testing, the specimens were loaded at 2 N/s in translation direction until implant failure.

**Results:**

Formalin fixed specimen: Mean load failure was 513.8 (±86.74) Newton (N) for group A (LMS) and 570.4 (±156.5) N for group B (CPS). There was no significant difference (*P* = .6905).

Fresh frozen specimen: Mean load failure was 402.3 (±96.4) N for group A (LMS) and 500.7 (±190.3) N for group B (CPS). There was no significant difference (*P* = .4206).

**Conclusion:**

In our loading model respecting the translational injury pattern and a flexion movement we could not verify statistically significant differences between lateral mass screws and cervical pedicle screws. Mean loading failure was slightly higher in the CPS group though.

## 1 Introduction

More than 50% of cervical injuries are located between C5 and C7.^[^1^,^^2^^]^ Especially translational injuries (Type C regarding AO-Classification) of the subaxial cervical spine are highly unstable and need to be treated by surgical stabilization.^[3]^ In case of internal fixation various techniques of posterior stabilization of the subaxial cervical spine are available. Lateral mass screws (LMS) are widely used.^[4]^ However, recently cervical pedicle screws (CPS) seem to be an alternative since computer-assisted navigated systems are available avoiding major complications like injuries of the spinal cord, vertebral arteries, and nerve roots.^[5]^ Already 1994 the clinical application for injuries of the subaxial cervical spine was described by Abumi et al.^[6]^ However, with regard to the risk of neurovascular lesions, the use of CPS remain controversial.^[4]^

Despite CPS have shown better stability in biomechanical studies^[^7^–^11^]^ concerns regarding the complication risks remain. Yoshihara et al^[4]^ described in their literature analyses significant higher vertebral injuries using CPS comparing to LMS whereas LMS were associated with loss of reduction, pseudarthrosis, and higher rate of screw loosening, not significant though. Even using computer-assistance Ludwig et al^[12]^ describe a screw failure rate of 24% for cervical pedicle screws in a cadaveric study.

In total biomechanical studies comparing LMS and CPS are in limited number and mainly describe stiffness and pullout strength.^[11]^ Till now there exist only a few biomechanical studies simulating translational injury model.

Aim of the present biomechanical study was to evaluate primary stability of LMS comparing CPS in a translational injury model.

## 2 Materials and methods

The biomechanical study was approved by the ethics committee (AZ 126/17, University Hospital Marburg, December 11, 2017). Consent of the anatomical donation for the purposes of medical research was obtained written from the donors.

Ten formalin fixed cervical spines were used to establish the injury model and loading protocol. Afterwards 10 fresh frozen intact cervical spines (C4-T1) were used. Computed tomography (CT) scans were performed before the procedure to exclude bone abnormalities. Dual-energy X-ray absorptiometry scans were performed prior to the study to measure bone mineral density (BMD).

Specimens were stored at –20°C, were thawed at room temperature overnight prior the study and were kept moist during all of the procedures using NaCl soaked wound dressings.

To assure comparability of the groups, spines were matched according to size and BMD and subsequently randomized into 2 groups. All tissues except ligaments and discs were dissected from the cervical spines and then T1 was embedded in cold-curing resin for surface testing and impressions (Technovit 3040, Kulzer, Germany). All dissections were performed by a medical student (J.R.) who was trained on formalin fixated spine segments that were used to establish the preparation and loading protocol.

To simulate a translational injury with complete intervertebral disc-ligament rupture and facet dislocation according to the AO-Classification (Type C) we used an already in the laboratory established injury model.^[13]^ The entire posterior and anterior ligament complex between C5 and C6 was cut (complete vertebral dissection). After separating the vertebral bodies, the discs were completely removed and the facet joints were resected.

**Figure 1:**
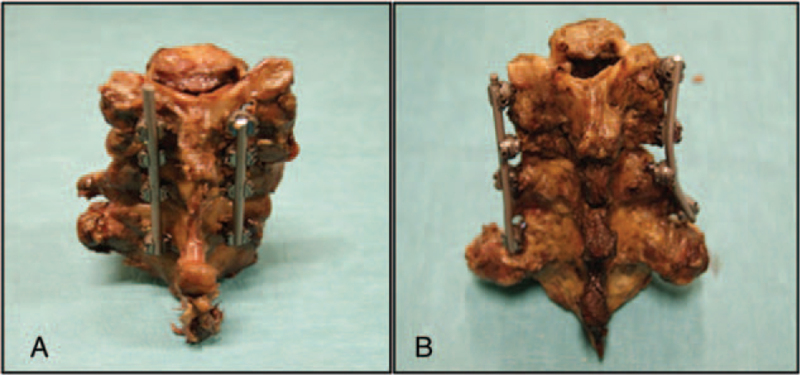
Preparation of cadaver spines: A = posterior stabilization with lateral mass screws (LMS) in group A, B = posterior stabilization with cervical pedicle screws (CPS) in group B.

Dorsal bilateral stabilization of C 4/C 5 to C 6/C 7 was performed in group A by LMS, in group B by cervical pedicle screws (CPS) using the MESA Mini Spinal System (K2M, USA) (see Fig. 1).

**Figure 2:**
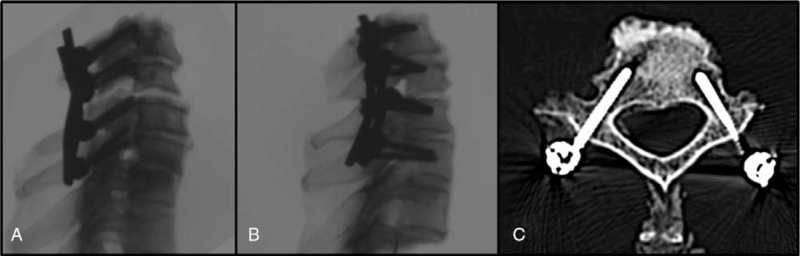
Post-procedure X-rays and CT: A = lateral mass screws (group A), B = cervical pedicle screws (group B), C = post-procedure CT of cervical pedicle screws (group B).

Visual inspection and radiological control with fluoroscopy were performed during inserting the screw to avoid dislocation of the screw. Screw position was controlled by postoperative CT scans (see Fig. 2).

**Figure 3:**
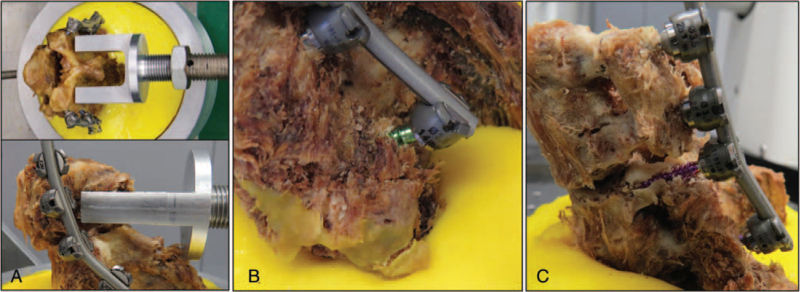
Spine loading: A = loading force positioned at the C4/C5 facet joint to produce a flexion movement, B = mean load failure in group A (implant failure of a lateral mass screw), C = mean load failure in group B (implant failure of a cervical pedicle screw).

The sample was placed in the loading jig. The force vector was positioned at the C4/C5 facet joint to produce a flexion movement (see Fig. 3). Continuous loading was performed using a material testing machine (Instron 5566) at 2 N/s. During testing, a load displacement curve was generated and the mean load failure was measured when the force changed from a positive to a negative value on the load displacement curve (see Fig. 3).

The measured values are given in mean and standard deviation and were compared statistically using the Mann–Whitney *U* test (GraphPad Prism 5.03, GraphPad Software Inc.; San Diego, USA). Statistical significance was assigned at a probability level of less than .05.

## 3 Results

A translational injury was successfully created in all of the cadaver cervical spines. There were no complications or technical problems.

### 3.1 Formalin fixed specimen

BMD was 0.703 (±0.094) for group A (LMS) and 0.682 (±0.080) for group B (CPS). There was no significant difference (*P* = .8413)

Specimen size was 110.22 (±12.22) mm for group A and 109.68 (±9.06) mm for group B. No significant difference was found (*P* = 1.0000).

CT scans after dorsal stabilization showed a correct screw position in all specimens.

Mean load failure was 513.8 (±86.74) Newton (N) for group A (LMS) and 570.4 (±156.5) N for group B (CPS).

**Figure 4:**
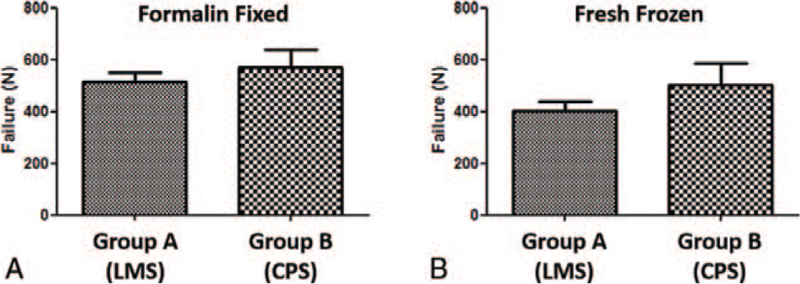
Mean load failure in Newton; A = formalin fixed specimen, Group A = lateral mass screws (LMS), Group B = cervical pedicle screws (CPS), B = fresh frozen specimen, Group A = lateral mass screws (LMS), Group B = cervical pedicle screws (CPS).

There was no significant difference between both screw fixation techniques (*P* = .6905) (see Fig. 4).

### 3.2 Fresh frozen specimen

BMD was 0.650 (±0.107) for group A (LMS) and 0.652 (±0.160) for group B (CPS). There was no significant difference (*P* = .8413)

Specimen size was 123.2 (±10.47) mm for group A and 122.4 (±9.29) mm for group B. No significant difference was found (*P* = .9166).

CT scans after dorsal stabilization showed a correct screw position in all specimens.

Mean load failure was 402.3 (±96.4) N for group A (LMS) and 500.7 (±190.3) N for group B (CPS).

There was no significant difference between both screw fixation techniques (*P* = .4206) (see Fig. 4).

## 4 Discussion and conclusions

More than 50% of cervical injuries are located between C5 and C7.^[^1^,^^2^^]^ Especially translational injuries (Type C regarding AO-Classification) of the subaxial cervical spine are highly unstable and need to be treated by surgical stabilization.^[^3^,^^14^^]^ Translation injury mechanisms lead to significant ligament disruption. Vaccaro et al^[15]^ proposed the Subaxial Injury Classification (SLIC) scoring system which takes into account injury morphology, integrity of the disc-ligament soft tissue complex and neurological status. Translation and rotation injuries are assigned the greater number of points on the morphological scale (4 points). Since these injuries are typically associated with ligament disruption (2 points), the SLIC injury score reaches already 6 points before the neurological status is taken into consideration. If the patient has a score of 5 points or more, surgical treatment is recommended. Recently the new AOSpine classification for subaxial cervical spine injuries and the corresponding treatment recommendations were published.^[^3^,^^16^^]^ Translatory instability including the disruption of the anterior and the posterior tension band is classified as type C injury and urgent surgical stabilization is recommended.^[3]^ Various surgical strategies have been described in various studies using posterior, anterior or posterior-anterior fixation.^[^1^,^^17^^,^^18^^]^

Nevertheless, surgical strategies have not been precisely established. There is still a lack of guidance for using anterior, posterior or combined surgical procedures.^[19]^ Whereas clinical issues support anterior surgery regarding decompression of the spinal cord, a minor surgical trauma and fusion ability, biomechanical studies showed better stability for posterior instrumentation.^[^1^,^^20^^–^24^]^ Due to biomechanical results 360° treatment seems to offer the best stability.^[7]^

Various techniques of posterior stabilization of the subaxial cervical spine are available. Lateral mass screws (LMS) are widely used.^[4]^ However, recently cervical pedicle screws (CPS) seem to be an alternative since computer-assisted navigated systems are available avoiding major complications like injuries of the spinal cord, vertebral arteries and nerve roots.^[^5^,^^18^^]^ Already 1994 the clinical application for injuries of the subaxial cervical spine was described by Abumi et al.^[6]^ However, with regard to the risk of neurovascular lesions, the use of CPS remain controversial.^[4]^ Despite CPS have shown better stability in biomechanical studies^[^7^–^11^]^ concerns regarding the complication risks remain. Yoshihara et al^[4]^ described in their literature analyses significant higher vertebral injuries using CPS comparing to LMS whereas LMS were associated with loss of reduction, pseudarthrosis and higher rate of screw loosening, not significant though. Even using computer-assistance Ludwig et al^[12]^ describe a screw failure rate of 24% for cervical pedicle screws in a cadaveric study. In total biomechanical studies comparing LMS and CPS are in limited number and mainly describe stiffness and pullout strength.^[11]^ Till now there exist only a few biomechanical studies simulating translational injury model.

Aim of the present biomechanical study was to evaluate primary stability of LMS comparing CPS in a translational injury model type C regarding AOSpine classification system. To simulate a spinal flexion movement, continuous loading was applied sagittal dorsal on the C4/C5 facet joints. The load was applied continuously at 2 N/s until the force changed from a positive to a negative value on the load displacement curve (mean failure load). Our results suggest that mean load failure of cervical pedicle screws (CPS) was higher than mean load failure of lateral mass screws (LMS) without significant difference though.

Comparative biomechanical studies already proved higher stability of CPS in cyclic loading and pullout models.^[^8^,^^10^^,^^11^^]^ The results of Ito et al^[11]^ suggest significant higher pullout strength of CPS comparing to LMS. Kothe et al^[10]^ showed significant better stability in cyclic loading in favor of CPS comparing to LMS. Johnston et al^[8]^ demonstrated a significant lower rate of screw loosening as well as higher stability in fatigue testing for CPS comparing to LMS.

In the present study we performed continuous loading using a flexion movement model instead of pullout or cyclic loading model in order to detect primary stability regarding flexion motion. In this model we could not prove statistically significant differences between CPS and LMS. Reason of these findings are probably the degree of screw insertions since the LMS, which were inserted via the Magerl technique, have an advantageous angle to the force vector we applied.

Our study has some limitations. All muscles and soft tissues except the ligaments were removed; soft tissue could be important to simulate more physiologic conditions. Furthermore, continuous loading was performed sagittal dorsal to achieve cervical spine flexion only without rotation. Despite these limitations, the study showed comparable results with regard to biomechanical stability. To our knowledge, this is the first biomechanical study using this kind of loading model for posterior instrumentation respecting the translational injury pattern and flexion movements.

Despite the superiority of cervical pedicle screws in prior biomechanical studies regarding pullout and fatigue testing the lateral mass screws remain a solid posterior stabilization regarding primary stability. In our loading model respecting the translational injury pattern and a flexion movement we could not verify statistically significant differences between lateral mass screws (LMS) and cervical pedicle screws (CPS). Mean loading failure was slightly higher in the CPS group though. Respecting the possible complications and difficult application of CPS this screw technique represents an alternative method with comparable biomechanical quality regarding the primary stability comparing to lateral mass screws for cervical posterior stabilization. Prospective randomized studies comparing CPS and LMS are needed to verify the biomechanical findings and investigate clinical outcome values.

## Author contributions

AK, LO, and SR have designed the study and have prepared the test. The work in the laboratory and preparation have been mainly performed by AK, LO, JR, TK, CB, and MB. Data acquisition and validation by TK, LO, MB, CB, and AK. Preparing and correction of the manuscript by all authors mainly AK, JR, and LO.

All authors have read and approved the manuscript.

**Conceptualization:** Ludwig Oberkircher, Steffen Ruchholtz, Antonio Krüger.

**Data curation:** Ludwig Oberkircher, Julia Riemenschneider, Martin Bäumlein, Tom Knauf, Christopher Bliemel, Antonio Krüger.

**Formal analysis:** Ludwig Oberkircher, Julia Riemenschneider, Martin Bäumlein.

**Investigation:** Ludwig Oberkircher, Julia Riemenschneider, Martin Bäumlein, Tom Knauf, Christopher Bliemel, Antonio Krüger.

**Methodology:** Ludwig Oberkircher.

**Project administration:** Ludwig Oberkircher.

**Supervision:** Ludwig Oberkircher, Steffen Ruchholtz.

**Validation:** Ludwig Oberkircher.

**Writing – original draft:** Ludwig Oberkircher, Julia Riemenschneider, Antonio Krüger.

**Writing – review & editing:** Martin Bäumlein, Tom Knauf, Christopher Bliemel, Steffen Ruchholtz.
